# New OPTN Simultaneous Liver-Kidney Transplant (SLKT) Policy Improves Racial and Ethnic Disparities

**DOI:** 10.3390/jcm9123901

**Published:** 2020-12-01

**Authors:** Daniela Goyes, John Paul Nsubuga, Esli Medina-Morales, Vilas Patwardhan, Alan Bonder

**Affiliations:** 1 Department of Medicine, Loyola Medicine—MacNeal Hospital, Berwyn, IL 60402, USA; daniela.goyesvaca@luhs.org; 2 Department of Medicine, Beth Israel Deaconess Medical Center, Boston, MA 02215, USA; jnsubuga@bidmc.harvard.edu; 3 Division of Gastroenterology and Hepatology, Beth Israel Deaconess Medical Center, Boston, MA 02215, USA; jemedina@bidmc.harvard.edu (E.M.-M.); vpatward@bidmc.harvard.edu (V.P.)

**Keywords:** simultaneous liver-kidney transplant, disparities, post-graft survival

## Abstract

(1) Background: On 10 August 2017, the Organ Procurement and Transplantation Network (OPTN) adopted standardized eligibility criteria to properly determine which transplant candidates should undergo Simultaneous Liver-Kidney Transplant (SLKT). Racial and ethnic disparities have not been examined after 2017. Therefore, using the United Network for Organ Sharing (UNOS), we aim to evaluate post-graft survival outcomes among Caucasians, African Americans, and Hispanics. (2) Methods: Kaplan–Meier curves and Cox regression models are used to compare post-transplant graft survival for Caucasians, African Americans (AAs), and Hispanics. Competing risk analysis is used to evaluate the cumulative incidence of death or re-transplantation with re-transplantation and death as competing risks. (3) Results: On multivariate Cox regression analysis, no differences in graft survival are found in AA (hazard ratio (HR): 1.30; 95% CI: 0.74–2.29 *p* = 0.354) or Hispanics (HR: 1.18; 95% CI: 0.70–2 *p* = 0.520) compared to Caucasians after 2017. On competing risk analysis of the risk of death with re-transplantation as a competing risk, no difference is found between ethnic minorities after 2017. There is a similar finding from competing risk analysis of the risk of re-transplantation with death as a competing risk. (4) Conclusion: After introducing standardized eligibility criteria for SLKT allocation, the post-graft survival outcomes remain similar between the different racial and ethnic groups, displaying the benefits of adopting such policy in 2017.

## 1. Introduction

Since the implementation of the model for end-stage liver disease (MELD) score, there has been a significant increase in the number of simultaneous liver-kidney transplants (SLKTs) [1,2]. However, due to the absence of a kidney allocation system in the context of liver transplant (LT), the practice of SLKT was unclear [3]. Regardless of the time spent by a candidate on the waitlist, the Organ Procurement and Transplantation Network (OPTN) prioritized candidates seeking a SLKT before candidates who were listed only for a kidney when the liver candidate and the deceased donor were in the same donation service area (DSA) [4]. As survival outcomes for LT recipients depend also on obtaining a kidney transplant, the regional SLKT allocation created a tremendous concern for the LT community [3]. A high percentage of SLKT patients received better quality kidneys than patients receiving kidney transplants alone [5]. These practices were criticized because they went against the OPTN Final Rule [3] and due to the concern about the unequal allocation of organs.

To address the previously mentioned concerns, on 10 August 2017, the OPTN adopted standardized eligibility criteria to properly determine which transplant candidates should undergo SLKT. This new policy established that the candidate would no longer receive priority for an SLKT at the time of their liver offer unless the candidate met certain medical eligibility criteria that suggested kidney dysfunction. The new criteria amended those originally proposed in 2009 [3]. It is defined by the presence of (1) chronic kidney disease with a measured or calculated estimated glomerular filtration rate (eGFR) ≤ 35 mL/minute as opposed to previous criteria that suggested eGFR < 30 mL/minute, (2) a sustained acute kidney injury without a specific number of dialysis requirements (previously two dialyze/week), or (3) the presence of metabolic diseases such as hyperoxaluria, atypical hemolytic uremic syndrome from mutations in factor H and possibly factor I, familial non-neuropathic systemic amyloidosis, or methylmalonic aciduria [6]. This policy also includes a safety net for patients who did not recover renal function after LT or those who consequently developed renal dysfunction [6]. Before the implementation of the new SLKT criteria, studies had noted disparities among ethnic minorities that directly affected post-graft survival rates [7]. However, racial and ethnic disparities have not been examined after 2017. Even though the amended SLKT criteria did not attend to eliminate disparities among racial/ethnic minorities, the transplant community has a responsibility to evaluate if allocation policy changes have triggered or exacerbated preexisting disparities. Therefore, using the United Network for Organ Sharing (UNOS), we aimed to evaluate post-graft survival outcomes among Caucasian, African American (AA), and Hispanic patients to assess whether racial/ethnic disparities are present in the post-SLKT era.

## 2. Materials and Methods

### 2.1. Study Population

The UNOS OPTN database was used to identify Caucasian, AA, and Hispanic patients who underwent SLKT from 10 August 2017 to 31 December 2019. We excluded children (age < 18 years), patients listed as status 1, and living donor transplants.

### 2.2. Outcomes

The primary outcome was post-transplant graft survival, defined as a composite of post-transplant death or need for re-transplantation. Secondary outcomes included post-transplant death alone or the need for re-transplantation alone.

### 2.3. Statistical Analysis

Demographic and clinical characteristics of patients who underwent SLKT after 2017 were compared using the Kruskal–Wallis test for continuous variables and Pearson’s chi-squared test (χ^2^) for categorical variables.

Continuous variables are reported as a median interquartile range (IQR), and categorical variables are summarized using percentages. Kaplan–Meier curves and Cox regression models were used to compare post-transplant graft survival for Caucasians, AAs, and Hispanics. We adjusted for recipient characteristics including age at transplant, sex, ethnicity, body mass index at transplant, and model for end-stage liver disease (MELD) score at transplant and UNOS region. We also adjusted for graft characteristics, including cold ischemia time, donor age, and degree of ABO matching. Competing risk analysis was used to evaluate the cumulative incidence of death or re-transplantation with re-transplantation and death as competing risks. All statistical analyses were conducted using Stata version 14.0 (StataCorp LP, College Station, TX, USA).

## 3. Results

Between 10 August 2017 and 31 December 2019, a total of 1214 patients underwent SLKT in the United States. Baseline clinical and demographic characteristics divided by race/ethnicity are displayed in Table 1. Caucasian recipients accounted for the majority (67%) of the population. A greater proportion of patients were men across the groups, and there was no difference in terms of age. Hispanics presented more severe disease and had a higher MELD score at transplant than Caucasian and AA patients. Patients with non-alcoholic steatohepatitis (NASH) and alcohol-related liver disease (ALD) formed a larger proportion of the subjects undergoing SLKT in both Caucasians and Hispanics, while hepatitis C virus (HCV) was the most common disease present in AAs.

### 3.1. Graft Survival

**Figure 1:**
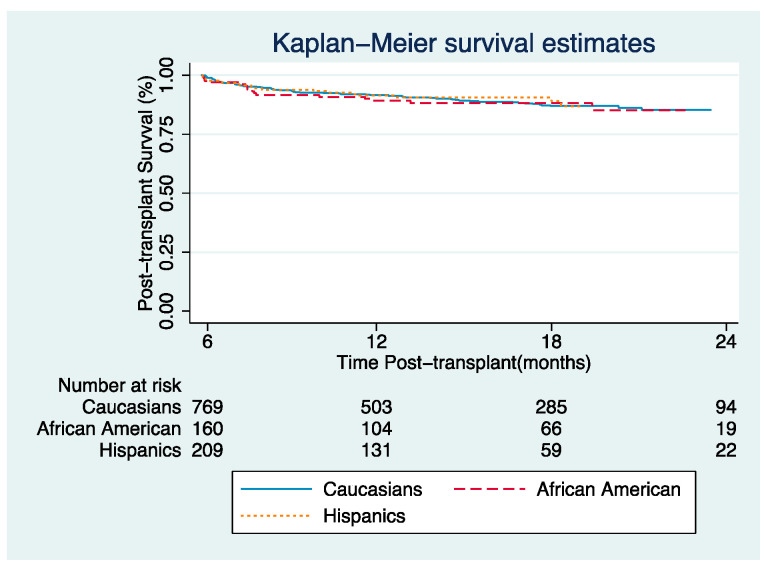
Unadjusted Kaplan–Meier estimates comparing graft survival (composite of post-transplant death and re-transplant by race/ethnicity).

There was no difference between racial/ethnic groups in post-transplant graft survival at six months, one year, and two years (*p* = 0.905) (Figure 1). On multivariate Cox regression analysis, being male, cold ischemia, ALD, and HCV were associated with a higher risk of graft failure. However, no differences in graft survival were found in AAs (hazard ratio (HR): 1.30; 95% CI: 0.74–2.29 *p* = 0.354) or Hispanics (HR: 1.18; 95% CI: 0.70–2 *p* = 0.520) compared to Caucasians after 2017 (Table 2).

### 3.2. Competing Risk Analysis

**Figure 2:**
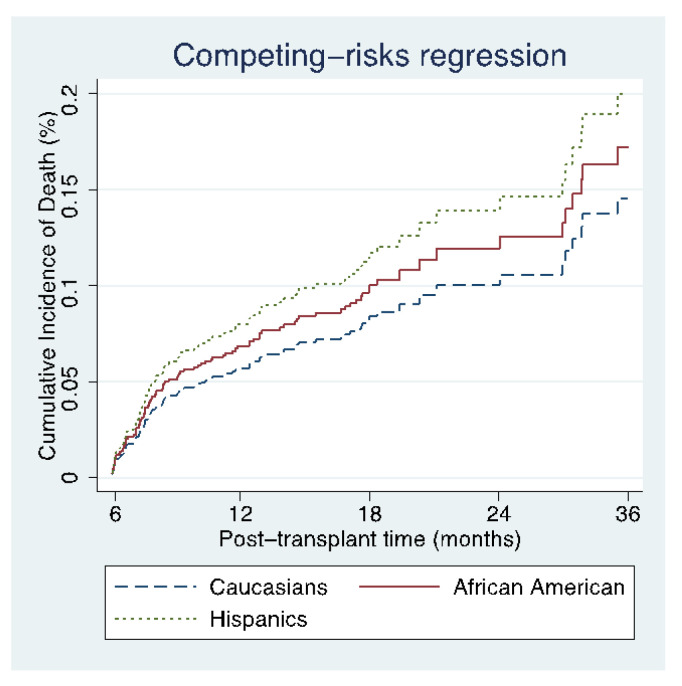
Cumulative incidence of death post-transplant (with re-transplant as competing risk) by race/ethnicity.

For competing risk analysis of the risk of death with re-transplantation as a competing risk, there was no difference between ethnic minorities after 2017 (AA subdistribution hazard ratio (SHR) 1.20, 95% CI 0.64–2.23; Hispanics SHR 1.42, 95% CI 0.83–2.41) compared to Caucasians (Table 3, Figure 2). Similarly, for the competing risk analysis of the risk of re-transplantation with death as a competing risk, we found no difference between AAs (SHR 3.36, 95% CI 0.77–14.6) and Hispanics (SHR 0.40, 95% CI 0.07–2.17) compared to Caucasians (Table 3).

## 4. Discussion

Although studies evaluating racial/ethnic disparities in SLKT are scant, several studies have demonstrated persistent disparities among ethnic minorities in post-liver-transplant survival outcomes [8,9]. In our study, we evaluated post-transplant graft survival outcomes among AA, Hispanic, and Caucasian patients, and found no differences in graft survival, risk of death, or re-transplantation among the three groups after the amendment of the SLKT allocation criteria. To the best of our knowledge, this is the first study to assess potential racial/ethnic disparities in the post-SLKT era.

Irrespective of differences across Caucasians, AAs, and Hispanics that may suggest more severe disease such as higher MELD score and, therefore, an expected greater proportion of liver graft losses and death, our study shows that the amended SLKT allocation policy is providing equitable access to transplants. Several factors could explain our results. First, promoting a system where organs are allocated equitably based on objective level of medical needs allows the most vulnerable patients to receive life-saving organs regardless race/ethnicity or socioeconomic status. Second, access to centers with high transplant volume, improved surgical expertise, ancillary care and support can play a role in better transplant outcomes. For example, Macomber et al. found a decreased mortality and improved post-LT outcomes especially in sicker patients at centers with high volume transplants [10]. Third, better patient compliance with immunosuppressive medication and post-transplant follow-up could also help lead to improved post-transplant outcomes as suggested by Taber et al. This study showed that nonadherence to post-transplant follow-up appointments was a risk factor for graft loss and death in kidney transplant recipients [11].

Our results are similar to those previously published by Chang et al. in 2019. Using the OPTN database, they compared all-cause mortality between Caucasians, AAs, and Hispanics, before the SLKT criteria amendment and found that AAs had a lower mortality risk before 24 months but had a higher mortality risk afterward; in contrast, Hispanic patients had a lower overall mortality risk than Caucasians [7]. Unfortunately, there are insufficient data before the SLKT criteria amendment to compare disparities across the two eras. Given the new implementation of the SLKT allocation policy, we were unable to report long-term outcomes after transplantation, which makes a direct comparison between the two studies difficult.

Our study also highlights the high number of conducted SLKT, despite the kidney organ shortage [12,13]. This result can be explained by the implementation of new policies such as the MELD score due to the inclusion of serum creatine in the scoring system, which is an independent predictor of post-LT outcomes [14]. Likewise, the adoption of Share-35, which prioritizes sicker patients for broader regional sharing, has increased the proportion of LT from 18.5% to 26.5% [15]. Additionally, NASH afflicts a great proportion of our study population and it has been associated with a higher prevalence and incidence of chronic kidney disease [16]. Therefore, these factors may contribute to a rising absolute number and proportion of SLKTs.

SLKT has a higher number of postoperative complications than kidney after liver transplantation (KALT). International organizations such as Eurotransplant, which facilitates patient-oriented organ allocation for eight member states of the European Union, are more conservative and favor sequential KALT [17]. There is data that support this approach, for instance in a study conducted by Bacarro et al. [18], who compared post-transplant outcomes between SLKT and LT, a higher incidence of acute renal failure was found during hospitalization in patients who underwent SLKT compared to the LT group (55% vs. 35%). During the first six months of follow-up, this incidence rose to 65% and 70%, respectively. Other complications such as infections, shock, and the need for blood transfusions were also higher in the SLKT group. However, data on the benefit of SLKT over KALT have been inconsistent. Simpson et al. [19] found an increased incidence of chronic rejection and a decreased half-life of renal allografts in KALT patients compared to SLKT patients. Superior liver allograft and patient survival rates were found in patients undergoing SLKT, especially in the context of hepatorenal syndrome in a study by Fong et al. [20]. Another study found that SLKT conferred a reduction in the risk of liver graft loss only in patients with long-term dialysis [21]. However, Tanriover et al., after stratifying patients according to the level of renal dysfunction and dialysis status at transplant, found that the survival benefit of SLKT was limited to patients with serum creatine ≥2 mg/dL and not on dialysis [21]. Nevertheless, our results demonstrate that the increase in the number of SLKT has not resulted in decreased graft survival or increased racial/ethnic disparities as previously stated.

The strength of our study is the use of a large-scale database of transplant recipients, which allowed our findings to reflect nationwide trends in SLKT. However, the lack of granularity inherent in a large nationwide database could limit our results given unmeasurable confounding variables. Furthermore, we were unable to assess cause of post-transplant death due to the amount of missing data.

## 5. Conclusions

During the pre-SLKT era, racial/ethnic disparities were common due to the absence of well-defined allocation criteria. After introducing standardized eligibility criteria for SLKT allocation, the post-graft survival outcomes remained similar between the different racial and ethnic groups, displaying the benefits of adopting such a policy in 2017. Nevertheless, disparities may arise later. Thus, the transplant community has a responsibility to continuously evaluate allocation policy changes to detect triggers or exacerbations of preexisting disparities and assess whether fair organ allocation is being made.

## Figures and Tables

**Table 1:** Baseline demographic and clinical characteristics of patients who underwent SLKT grouped according to ethnicity (*n* = 1214).

Variable	Caucasian *n* = 816 (67)	AA *n* = 172 (14)	Hispanic *n* = 226 (19)	*p*-Value
Sex (male), *n* (%)	520 (64)	94 (55)	123 (54)	0.009
Age, median (IQR)	59 (52–65)	58 (52–62)	58 (52–63)	0.077
BMI (IQR)	28 (25–33)	27 (24–31)	27 (25–32)	0.139
Primary diagnosis, *n* (%)				<0.001
NASH	261 (32)	18 (10)	73 (32)	
ALD	290 (36)	19 (11)	79 (35)	
HBV	6 (1)	4 (2)	2 (1)	
HCV	89 (11)	75 (44)	33 (15)	
AIH	12 (2)	5 (3)	5 (2)	
Cholestatic liver disease	26 (3)	12 (7)	2 (1)	
Other	130 (16)	39 (23)	32 (14)	
Cold ischemia, median (IQR)	6 (5–7)	6 (5–7)	6 (5–8)	0.018
ABO, *n* (%)				0.273
Matched	755 (93)	158 (92)	212 (94)	
Compatible	54 (7)	11 (6)	9 (4)	
Incompatible	7 (1)	3 (2)	5 (2)	
Donor age, median (IQR)	33 (25–45)	34 (27–46)	36 (24–48)	0.379
MELD score at transplant, median (IQR)	29 (24–35)	27 (22–34)	31 (23–36)	0.013
Private insurance, *n* (%)	398 (49)	52 (30)	76 (34)	<0.001
College education, *n* (%)	493 (60)	69 (40)	70 (30)	<0.001

AA: African American; IQR: interquartile range; BMI: body mass index; NASH: non-alcoholic steatohepatitis. ALD: alcohol-related liver disease; HBV: hepatitis B; HCV: hepatitis C; AIH: autoimmune hepatitis; MELD: model for end-stage liver disease.

**Table 2:** Multivariate Cox regression analysis of graft survival (composite of post-transplant death and re-transplantation).

Variable	HR	95% CI	*p*-Value	HR ^1^	95%CI	*p* Value
Sex (male)	1.30	0.89 to 1.89	0.171	1.53	1.03 to 2.29	0.035
Age	1.02	1 to 1.03	0.045	1.01	0.99 to 1.03	0.129
Race/Ethnicity						
Caucasian	Ref					
African American	1.15	0.69 to 1.90	0.574	1.30	0.74 to 2.29	0.354
Hispanics	1.06	0.66 to 1.70	0.791	1.18	0.70 to 2	0.520
BMI	0.98	0.95 to 1.01	0.296	0.95	0.92 to 0.99	0.21
Cold ischemia	1.10	1.05 to 1.15	<0.001	1.11	1.06 to 1.16	<0.001
ABO						
Matched	Ref					
Compatible	1.27	0.66 to 2.44	0.460	1.31	0.67 to 2.53	0.418
Incompatible	1.74	0.43 to 7.07	0.435	1.98	0.47 to 8.35	0.348
Donor age	1	0.99 to 1.02	0.3	1	0.99 to 1.02	0.382
MELD at transplant	1	0.98 to 1.03	0.466	1.01	0.98 to 1.03	0.313
Primary diagnosis						
NASH	Ref					
ALD	0.54	0.34 to 0.86	0.010	0.48	0.29 to 0.82	0.007
HBV	1.86	0.57 to 6	0.296	1.33	0.40 to 4.43	0.638
HCV	0.67	0.38 to 1.16	0.155	0.48	0.25 to 0.89	0.021
AIH	0.74	0.18 to 3.07	0.685	0.76	0.17 to 3.28	0.721
Cholestatic liver disease	0.69	0.25 to 1.94	0.492	0.51	0.17 to 1.50	0.227
Other	0.75	0.44 to 1.29	0.313	0.64	0.35 to 1.17	0.152

BMI: body mass index; NASH: non-alcoholic steatohepatitis; ALD: alcohol-related liver disease; HBV: hepatitis B; HCV: hepatitis C; AIH: autoimmune hepatitis; MELD: model for end-stage liver disease; HR: hazard ratio; CI: confidence interval; REF: reference. ^1^ Multivariate hazard ratio calculated from all variables in this table as well as UNOS region.

**Table 3:** Competing risk analysis.

Variable	Death with Re-Transplantation as Competing Risk			Re-Transplantation with Death as Competing Risk		
	SHR ^1^	95% CI	*p*-Value	SHR ^1^	95% CI	*p* Value
**Sex** (male)	1.54	0.99 to 2.38	0.052	1.58	0.53 to 4.63	0.403
**Age**	1.02	0.99 to 1.04	0.063	0.98	0.93 to 1.04	0.597
**Race/Ethnicity**						
Caucasian	Ref			Ref		
African American	1.20	0.64 to 2.23	0.557	3.36	0.77 to 14.6	0.106
Hispanics	1.42	0.83 to 2.41	0.193	0.40	0.07 to 2.17	0.293
**BMI**	0.95	0.91 to 0.99	0.028	0.97	0.88 to 1.06	0.533
**Cold ischemia**	1.12	1.08 to 1.16	<0.001	1.04	0.97 to 1.13	1.19
**ABO**						
Matched	Ref			Ref		
Compatible	1.38	0.69 to 2.76	0.361	0.96	0.12 to 7.60	0.975
Incompatible	1.16	0.14 to 9.3	0.886	4.98	1.01 to 24.5	0.048
Donor age	1	0.99 to 1.02	0.339	0.99	0.96 to 1.03	0.794
**MELD at transplant**	1.02	0.99 to 1.04	0.132	0.95	0.87 to 1.04	0.318
**Primary diagnosis**						
NASH	Ref			Ref		
ALD	0.53	0.29 to 0.95	0.034	0.35	0.10 to 1.15	0.085
HBV	1.59	0.48 to 5.2	0.438	1.68	3.96 to 7.10	<0.001
HCV	0.52	0.26 to 1.05	0.069	0.26	0.36 to 1.87	0.183
AIH	0.93	0.20 to 4.21	0.927	2.16	4.59 to 8.48	<0.001
Cholestatic liver disease	0.66	0.21 to 2	0.467	1.26	3.45 to 4.61	<0.001
Other	0.73	0.37 to 1.44	0.370	0.27	0.04 to 1.83	0.181

BMI: body mass index; NASH: non-alcoholic steatohepatitis; ALD: alcohol-related liver disease; HBV: hepatitis B; HCV: hepatitis C; AIH: autoimmune hepatitis; MELD: model for end-stage liver disease; HR: hazard ratio; CI: confidence interval; REF: reference. ^1^ Multivariate hazard ratio calculated from all variables in this table as well as UNOS region.
